# Implementing AI Models for Prognostic Predictions in High-Risk Burn Patients

**DOI:** 10.3390/diagnostics13182984

**Published:** 2023-09-18

**Authors:** Chin-Choon Yeh, Yu-San Lin, Chun-Chia Chen, Chung-Feng Liu

**Affiliations:** 1 Department of Plastic Surgery, Chi Mei Medical Center, Tainan 711, Taiwan; frankyeh1977@gmail.com (C.-C.Y.); plastylin@mail.chimei.org.tw (Y.-S.L.); chenjica@msa.hinet.net (C.-C.C.); 2 Department of Medical Research, Chi Mei Medical Center, Tainan 711, Taiwan

**Keywords:** burn patient, prognosis, prolonged hospital stay, skin graft needed, adverse complications, artificial intelligence, machine learning, hospital information systems

## Abstract

Background and Objectives: Burn injuries range from minor medical issues to severe, life-threatening conditions. The severity and location of the burn dictate its treatment; while minor burns might be treatable at home, severe burns necessitate medical intervention, sometimes in specialized burn centers with extended follow-up care. This study aims to leverage artificial intelligence (AI)/machine learning (ML) to forecast potential adverse effects in burn patients. Methods: This retrospective analysis considered burn patients admitted to Chi Mei Medical Center from 2010 to 2019. The study employed 14 features, comprising supplementary information like prior comorbidities and laboratory results, for building models for predicting graft surgery, a prolonged hospital stay, and overall adverse effects. Overall, 70% of the data set trained the AI models, with the remaining 30% reserved for testing. Three ML algorithms of random forest, LightGBM, and logistic regression were employed with evaluation metrics of accuracy, sensitivity, specificity, and the area under the receiver operating characteristic curve (AUC). Results: In this research, out of 224 patients assessed, the random forest model yielded the highest AUC for predictions related to prolonged hospital stays (>14 days) at 81.1%, followed by the XGBoost (79.9%) and LightGBM (79.5%) models. Besides, the random forest model of the need for a skin graft showed the highest AUC (78.8%), while the random forest model and XGBoost model of the occurrence of adverse complications both demonstrated the highest AUC (87.2%) as well. Based on the best models with the highest AUC values, an AI prediction system is designed and integrated into hospital information systems to assist physicians in the decision-making process. Conclusions: AI techniques showcased exceptional capabilities for predicting a prolonged hospital stay, the need for a skin graft, and the occurrence of overall adverse complications for burn patients. The insights from our study fuel optimism for the inception of a novel predictive model that can seamlessly meld with hospital information systems, enhancing clinical decisions and bolstering physician–patient dialogues.

## 1. Introduction

A burn injury refers to the damage to the skin or underlying tissues caused by exposure to heat, fire, chemicals, electricity, or radiation. Burn injuries can vary in severity, ranging from mild superficial burns to severe deep burns that can be life-threatening [1,2].

Burns are typically classified into different degrees based on their depth and severity:

First-degree burns: These are superficial burns that only affect the outer layer of the skin (epidermis). They usually result in redness, pain, and minor swelling, but do not typically cause blistering.

Second-degree burns: These burns affect both the outer layer of the skin (epidermis) and the underlying layer (dermis). They are characterized by blistering, severe pain, redness, and swelling.

Third-degree burns: These burns extend through all layers of the skin, damaging nerve endings and underlying tissues. They may appear white, charred, leathery, or blackened. Third-degree burns often result in numbness due to nerve damage and may require surgical intervention for treatment [3,4,5].

The severity of a burn injury can also be assessed using the “Rule of Nines”, which divides the body into different regions, each accounting for a specific percentage of the total body surface area (TBSA). This helps in estimating the extent of the burn and determining the need for specialized care [6].

Immediate first aid for burns typically involves removing the source of heat, cooling the burn with running cool (not cold) water, and covering it with a clean cloth or dressing. However, for more severe burns or burns involving critical areas such as the face, hands, feet, or genitals, immediate medical attention is essential.

Treatment for burn injuries often includes wound cleaning, the application of topical medications, pain management, and, in some cases, surgical procedures like skin grafting to promote healing [4]. Additionally, rehabilitation and long-term care may be necessary for individuals with extensive burns to regain functionality and manage potential complications such as scarring, contractures, and emotional trauma [7,8]. Furthermore, burns occurring in specific delicate regions, such as the eyelids or penis, pose significant challenges for healthcare practitioners in terms of both treatment and care [9].

However, the hospital faces limitations in its resources to effectively treat burns and scalds. This becomes particularly challenging when dealing with a diverse range of burn patients, including those who have been affected by indoor fires and outdoor dust storms, such as the colored power fire. Therefore, it is crucial to determine the severity of each individual’s condition and allocate appropriate medical resources accordingly. To address this urgency, the Baux score, also referred to as the “Baux index”, was developed by Dr. Jean Baux in 1960 [10].

The Baux score takes into account the patient’s age and the percentage of total body surface area (TBSA) affected by burns. It is calculated using the following formula:

Baux score = age (years) + burned area (%)

rBaux score = age (years) + burned area (%) + (17 × I)

In which: I = 1 if the patient suffered inhalation injury; and I = 0 if patients did not suffer inhalation injury.

For example, for a 35-year-old burn patient who suffered burns covering 20% of their total body surface area (TBSA), his/her Baux score and rBaux are:

Baux score = age + burned area = 35 + 20 = 55

rBaux score = age + burned area + (17 × I) = 35 + 20 + (17 × 1) = 35 + 20 + 17 = 72

The rBaux score ranges from 0 to 216. A higher Baux score indicates a greater risk of mortality. The score is used as a prognostic tool to assess the severity of burn injuries and help guide treatment decisions. Scoring systems like the Baux score and its variations provide a standardized method for assessing burn severity and predicting outcomes [11].

The rBaux scoring system has been widely and extensively utilized in clinical settings, garnering widespread acceptance for its effectiveness in predicting the likelihood of mortality in patients suffering from burns and scalds. According to Lam et al. [12], the revised Baux score has been found to be more accurate than the Baux score. However, they recommend its application solely for prognosis purposes in adult and elderly burn patients within developing countries [13,14]. Nevertheless, there have been studies indicating that the predictive power of the Baux score is limited, suggesting its inadequacy in accurately predicting burn outcomes. One such study conducted by Roberts et al. [15] (2012) found that the Baux score had a low sensitivity and specificity in predicting mortality in a cohort of burn patients in the US. Similarly, a study by Kirimi et al. [16] (2013) demonstrated that the Baux score exhibited poor discrimination in predicting complications such as infections and organ dysfunction.

Furthermore, alternative scoring systems, such as the Anesthesiologists Physical Status (ASA PS) Score [17] and body mass index (BMI) [18], are employed to forecast mortality in cases of burn injuries.

However, the aforementioned scoring methods can only provide limited clinical predictive information and may not effectively handle the variability of changing medical conditions and the diverse consultation requests from patients and their families, leading to a communication and information gap between healthcare providers and patients.

Moreover, the condition of severely burned patients typically exhibits rapid fluctuations, sometimes even on the scale of hours or minutes. Therefore, acquiring real-time clinical data, such as the information provided by Volumeview [19], and effectively integrating and interpreting these data, pose significant challenges and are of paramount importance. Consequently, we seek to harness the power of AI/ML to fully leverage this invaluable information, thereby making a valuable contribution to the enhancement of clinical care quality.

In view of this, it is urgent and necessary to develop more real-time and high-quality burn prediction tools to meet the requirements of modern precision medicine. Therefore, our study aims to develop a prediction model for high-risk burn patients and identify the factors that potentially increase the risk for mortalities and complications using AI/ML approaches based on a large database of burn patients in a Taiwanese center. We made a comparison of the prediction quality with the Baux score, and a prediction system based on our best model was implemented into practice as well.

## 2. Materials and Methods

### 2.1. Study Design, Setting, and Samples

**Figure 1:**
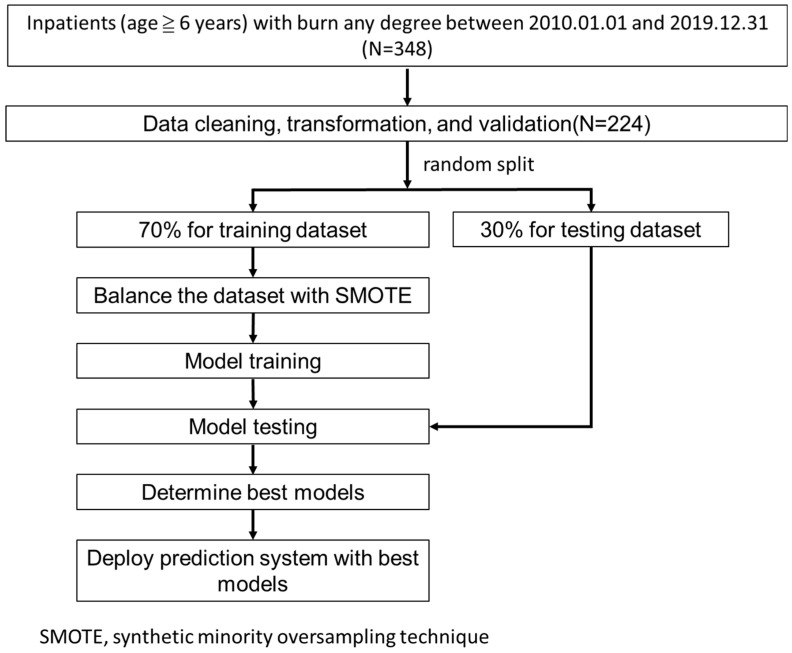
Research flow.

All inpatients with burn of any degree (ICD-9: 948.XX or ICD-10: T31.XX) in the first 6 diagnosis codes from 1 January 2010 to 31 December 2019 were included, but those aged ≤6 years old (5 cases) were excluded. Overall, 348 raw cases were used in the study. Figure 1 shows our research flow.

The study was approved by the Institutional Review Board of the Chi Mei Medical Center (IRB Serial No.: 11206-014). All methods were carried out following relevant guidelines and regulations. Informed consent from patients was waived due to the retrospective nature of the study.

### 2.2. Feature and Outcome Variables

We chose three outcome variables for the prediction models: (1) graft surgery (operation code 62015), (2) prolonged hospital stay (hospitalization days >14 days), and (3) overall adverse effects (sepsis, use of respirator, pneumonia, chronic kidney disease (CKD), mortality, and prolonged hospital stay).

Furthermore, we chose 12 feature variables, based on literature review and clinical experience, for building these prediction models:

(1) Basic information: gender, age, body mass index (BMI), smoking history, and escharotomy;
(2) Burn data: burn area, burn site—perineum, and burn site—extremities;
(3) Lab information: white blood cell (WBC), hemoglobin, creatinine, and glutamate pyruvate transaminase (GPT).

### 2.3. Model Building and Performance Evaluation

We used all the variables to build the prediction models to maximize model performance without performing any feature selection preprocessing. The data were randomly stratified into a training dataset (70%) and a testing dataset (30%). The SMOTE method (synthetic minority oversampling technique) [20] was used to deal with the data imbalance due to the fewer related positive classes (outcomes to be predicted, such as mortality) in the training dataset. The model of each outcome was built with 4 machine-learning algorithms, including (1) logistic regression, (2) random forest, (3) LightGBM, and (4) XGBoost.

We used a grid search with 5-fold cross-validation to build the best models based on the training dataset. We, then, used the testing dataset to evaluate the performance of the built models with indicators of accuracy, sensitivity, specificity, and AUC (area under the receiver operating characteristic curve).

### 2.4. Implementation and Deployment of the Best Models

The models with the highest AUCs were judged as the best and were used to implement a web-based prediction application and deployed into practice for physicians’ decision making. The web-based predictive application was developed with the Microsoft Visual Studio^®^ tool (v 17.7).

## 3. Results

### 3.1. Demographics

From 1 January 2010 to 31 December 2019, a total of 384 burn inpatients who are above 6 years old were enrolled in the study. After data cleaning and missing-value deletion, 224 cases underwent analysis. Overall, 70% of the data were randomly split for model training, and 30% for model evaluating.

Table 1, Table 2 and Table 3 show the demographics and characteristics of the patients with graft surgery, prolonged hospital stay (>14 days), and overall adverse effects, respectively. In total, the mean age was 45.8, and most patients were males (66.1%); about 50.4% of them were categorized as the least mild condition of burned area rank 1, while 18.8% of them were categorized as the most severe condition of burned area rank 4. The mean Baux score was 69.2.

### 3.2. Machine-Learning Modeling Results

The model performance of each predicted outcome was summarized in Table 4. In model of graft surgery, the highest AUC was found in the random forest model with a value of 0.757, followed by the logistic regression model, LightGBM model, and XGBoost model with values of 0.755, 0.745, and 0.738, respectively. In the model of prolonged hospital stay, the highest AUC was found in the XGBoost model with a value of 0.815, followed by the random forest model, LightGBM model, and logistic regression model with values of 0.801, 0.797, and 0.720, respectively (see Table 4). Finally, in the model of overall adverse effects, the highest AUC was found in the LightGBM model with a value of 0.845, followed by the logistic regression model, random forest model, and XGBoost model with values of 0.832, 0.822, and 0.816, respectively.

### 3.3. Interpreting the Feature Importance

**Figure 2:**
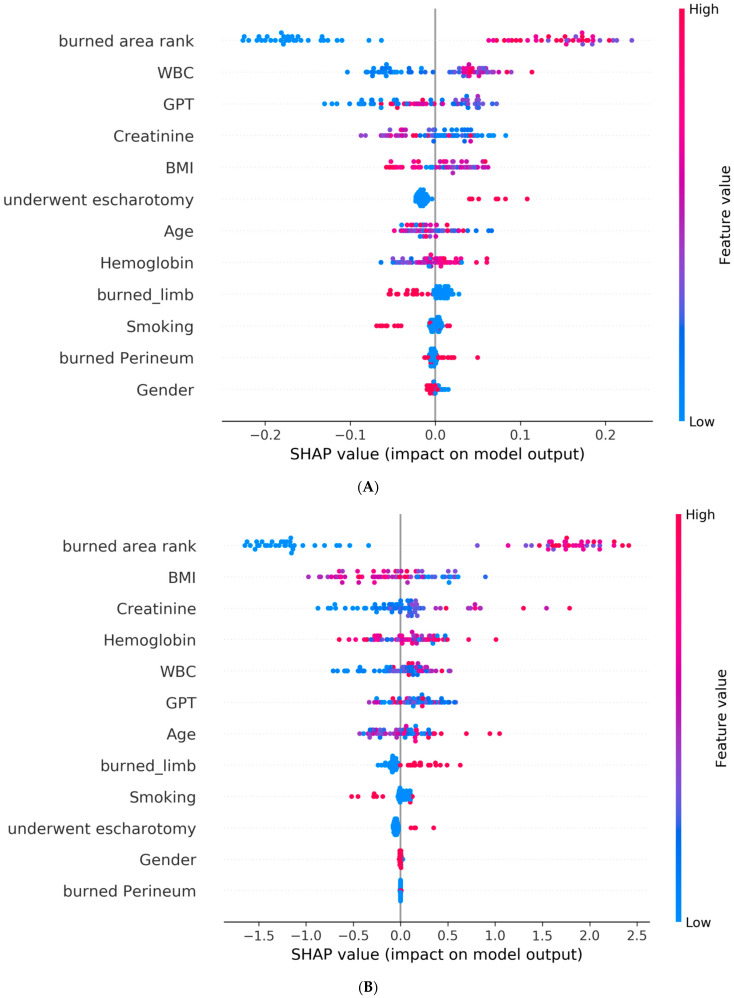

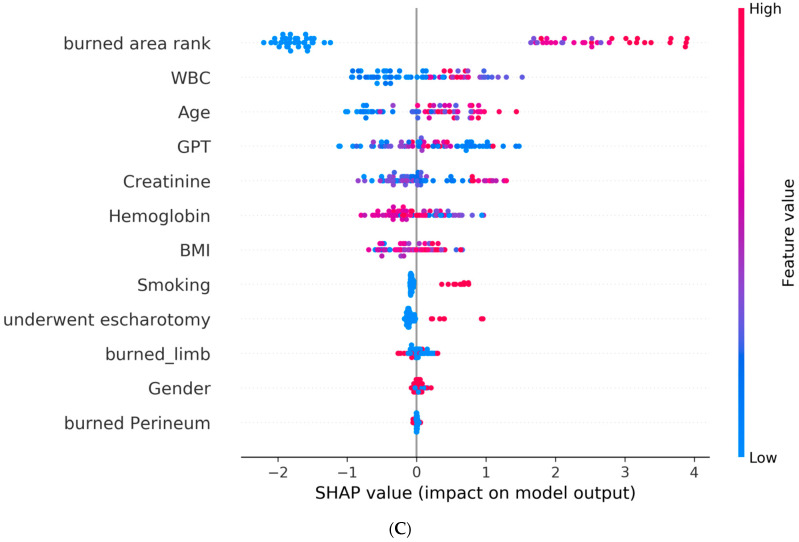
SHAP global explanation on the best model: (**A**) SHAP plot for model of graft surgery (random forest); (**B**) SHAP plot for model of prolonged hospital stay (XGBoost); (**C**) SHAP plot for model of overall adverse effects (LightGBM).

**Figure 3:**
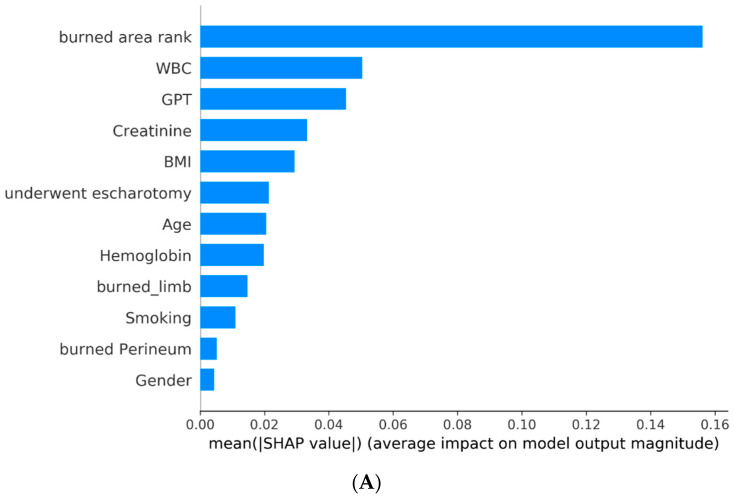

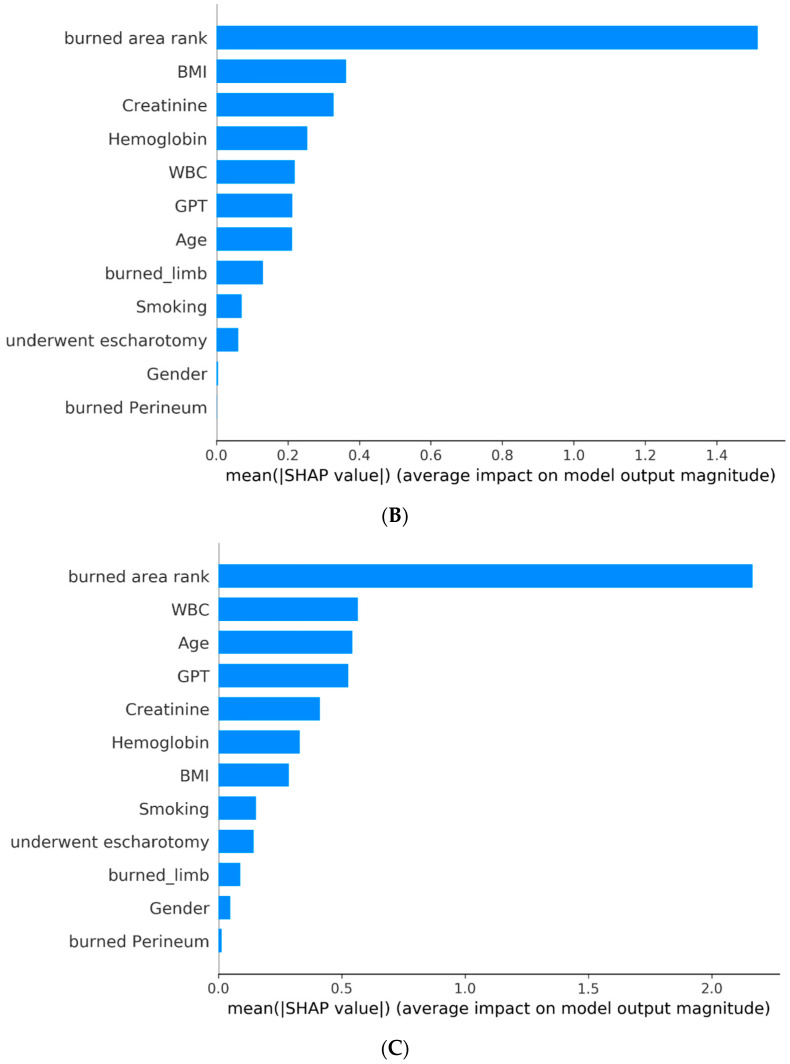
Absolute SHAP value of each feature on the best model: (**A**) SHAP plot for model of graft surgery (random forest); (**B**) SHAP plot for model of prolonged hospital stay (XGBoost); (**C**) SHAP plot for model of overall adverse effects (LightGBM).

Furthermore, for a better interpretation of how each feature contributes to the associated outcome, we performed a SHAP (SHapley Additive exPlanations) [21] analysis for each best AI model. In the SHAP global explanation plot, dots in red and blue indicate higher and lower feature values, respectively. A dot distribution to the left of the horizontal axis point 0 represents a negative correlation to the outcome, while a distribution to the right represents a positive correlation. The y-axis indicates the feature name, in order of importance from the top to the bottom of the plot. The wider the dots of the feature distributed, the greater the influence of the feature on the outcome. Figure 2 and Figure 3 depict global explanations of the best models and mean absolute SHAP values, respectively. As shown in Figure 2A, the x-axis represents the SHAP values of each of the features by which the model predicts the graft surgery. The interpretation of the summary plot shows that a higher burn area rank (higher values visible in red on the horizontal bar) implies an increase in the predicted outcome; conversely, a smaller burn area rank (visible in blue) is associated with a decrease in the predicted outcome. The feature of burn area rank was similarly explained in predicting a prolonged hospital stay and adverse effects. The same interpretation can be applied to the rest of the features.

Figure 3 shows the absolute SHAP value of each feature, presenting its importance on the associated outcome. It can be interpreted that the top three critical features on graft surgery are burned area rank, WBC, and GPT; the top three critical features on a prolonged hospital stay are burned area rank, BMI, and creatinine; and the top three critical features on overall adverse effects are burned area rank, WBC, and age.

### 3.4. Performance Comparison of AI Model and Baux Score

We compared the best AI-based model with the Baux score by the performance indicators. For this, we first calculated the indicators of accuracy, sensitivity, specificity, and AUC for AI models and Baux score models. We, then, performed the DeLong test for figuring out the significance of the model difference. As shown in Table 5, AI models have higher values for all indicators than Baux score models. AI models of prolonged hospital stay and overall adverse effects outperformed Baux score models in a statistically significant manner (*p* < 0.05).

### 3.5. Clinical Prediction Application Development and Deployment and User Preliminary Evaluation

**Figure 4:**
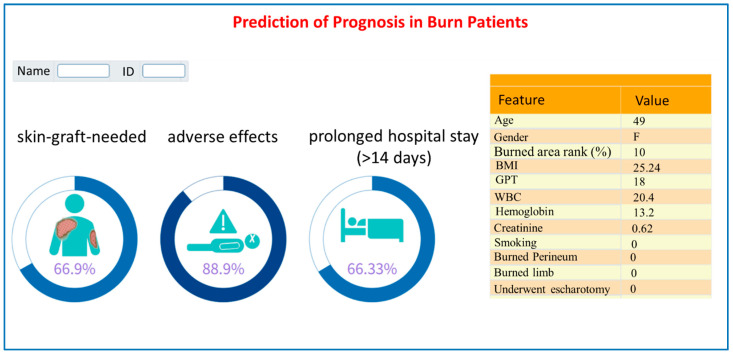
A snapshot of the prediction system.

For clarifying the feasibility and acceptance of our AI models, we developed an AI risk prediction system based on the three best models and deployed it in a burn critical care center for assisting with the physician’s decisions. Figure 4 showed a snapshot of the AI system (a probability ≥50% indicates a high probability of causing the adverse outcome). Models were built in the Python programming language and the Web-based interface was built in MS Visual Studio^®^ with VB (v 17.7).

We, then, demonstrated the AI system to the pilot burn care staff (three nurses and two physicians) and received positive feedback. They see this as a useful tool for the timely identification of high-risk burn patients. According to the risk probability, care staff can consider appropriate treatment plans to optimize the utilization of medical resources. It can greatly improve the quality and efficiency of burn patient care.

## 4. Discussion

In this study, we collected and analyzed a comprehensive set of laboratory data and clinical information to gain valuable insights. Our research encompassed crucial factors such as body mass index, gender, age, blood pressure, body temperature (BT), total body surface area (TBSA), hemoglobin levels, alanine transaminase levels, glucose levels, platelet count, blood urea nitrogen levels, creatinine levels, and more.

By including these significant parameters in our analysis, we aimed to capture a holistic view of the subjects and their health profiles. The diverse range of data points allowed us to explore the relationships between various variables and draw meaningful conclusions.

In summary, our study employed a robust dataset consisting of crucial laboratory measurements and clinical information. By considering these factors, we aimed to enhance the depth and accuracy of our findings, providing a comprehensive understanding of the subject population.

Moreover, we took into consideration several prevalent and significant comorbidities, including diabetes, hypertension, and cardiovascular disease. These conditions play a crucial role in determining patient outcomes.

We utilized all the aforementioned features to develop predictive models for various outcomes, such as in-hospital mortality, acute respiratory failure during hospitalization, ventilator dependence, renal failure, a prolonged hospital stay, and the need for skin grafting. To obtain the necessary data, we primarily relied on the routine emergency department (ED) charts and regular medical records. This approach eliminated the need for additional examinations while ensuring we had an ample amount of relevant data at our disposal.

By leveraging these readily available sources, we were able to capture a comprehensive range of information essential for our analysis. This streamlined approach allowed us to focus on utilizing the existing data to develop robust predictive models, saving time and resources without compromising the quality of our study.

This study stands out as one of the rare tools that aim to predict the severity of burn injuries in patients by leveraging diverse clinical characteristic data. By employing various statistical models and machine-learning approaches (i.e., logistic regression, random forest, SVM, KNN, LightGBM, ML, and Xgboost), we successfully achieved positive predictive outcomes. These results were anticipated, and we firmly believe that they hold significant value in terms of predicting and managing patients’ conditions effectively.

In the context of predicting the necessity of skin graft surgery using a machine-learning model (random forest), Figure 2A and Figure 3A indicate that the total body surface area (TBSA) has the most significant impact on the requirement for skin graft surgery, followed by the white blood cell count (WBC) and GPT (a liver enzyme), whereas gender has minimal influence. Higher values in the burn area rank correlate with an increased risk of undergoing skin graft surgery, indicating a greater likelihood of needing the procedure. Similarly, WBC levels also influence the risk of requiring skin graft surgery. Therefore, early and adequate fluid resuscitation is crucial in reducing the necessity of surgery.

Figure 2B and Figure 3B reveal that the total body surface area (TBSA) has the most significant impact on prolonged hospitalization, followed by the body mass index (BMI) and creatinine levels. The location of the burn or scald and gender have minimal influence. Higher TBSA values indicate a higher risk of prolonged hospitalization (exceeding 14 days). This suggests that obesity and impaired renal function are both risk factors associated with prolonged hospital stays.

Figure 2C and Figure 3C highlight the factors influencing different complications during hospitalization, as indicated by the LightGBM model. Notably, the total body surface area (TBSA) exhibits the most significant impact on overall adverse outcomes, followed by white blood cell count (WBC) and age. On the other hand, the location of the burn or scald and gender have minimal influence. Higher values in TBSA, WBC, and age are associated with an increased risk of overall adverse outcomes. These findings emphasize the importance of implementing specific preventive measures for elderly patients with extensive burn areas and a heightened risk of dehydration.

In Table 5, we compared the predictive results of the ML models we utilized (random forest, XGBoost, and LightGBM) with the Baux scoring in predicting the necessity of surgery, prolonged hospitalization, and the occurrence of complications. The ML models demonstrate superior performance over the Baux score in terms of accuracy, sensitivity, specificity, and AUC. Notably, the results for prolonged hospitalization and occurrence of complications exhibit significant differences based on the DeLong test.

The Baux score has traditionally been commonly employed to primarily predict the mortality probability of burn patients, while also implying the severity of the burn injury, with the result that it is of significant reference value and sees widespread use. However, our model, when compared to the Baux score, demonstrates superior performance in predicting prolonged hospital stays and complications. Consequently, it is expected to complement the shortcomings of the Baux score in clinical settings, thus offering a synergistic effect.

Next, we conducted a thorough review of several relevant and comparable studies. In Stylianou et al. [22]’s study, an established logistic mortality model was compared to machine-learning methods (artificial neural network, support vector machine, random forests, and naive Bayes) using a population-based (England and Wales) case-cohort registry. They presented the following findings: Random forests were marginally better for a high positive predictive value and reasonable sensitivity. Neural networks yielded slightly better prediction overall. Logistic regression gives an optimal mix of performance and interpretability.

Liu and colleagues [23] reviewed several databases such as MEDLINE, the Cochrane Database of Systematic Reviews, and ScienceDirect, and performed a citation review of relevant primary and review articles—the databases were searched for studies involving burn care/research and machine learning in the year 2015. The review conducted by Liu and colleagues highlighted the potential of machine-learning techniques in burn care and research. The studies reviewed in 2015 demonstrated the effectiveness of machine-learning algorithms in various aspects of burn care, including diagnosis, treatment planning, prognostic prediction, and wound assessment. The findings underscore the importance of continued research and development in this area, as the integration of machine learning holds great promise for enhancing patient outcomes and improving the overall quality of burn care.

A few years later, Huang et al. [24] conducted a systemic review in which thirty articles were included. Nine studies used machine learning and automation to estimate the percent total body surface area (%TBSA) burned, four calculated fluid estimations, nineteen estimated the burn depth, five estimated the need for surgery, and two evaluated scarring. In their conclusion, the utilization of machine learning as an adjunct for evaluating burn wound severity has demonstrated promising results in improving diagnostic accuracy. These techniques provide an objective approach by leveraging diverse data points to enhance the assessment process. However, it is crucial to conduct further research to validate and refine existing models, ensuring their clinical feasibility and applicability. The integration of machine learning into burn wound evaluation has the potential to advance the field, empowering clinicians with valuable insights and, ultimately, enhancing patient care.

We summarized the comparison of these works in Table 6.

In recent years, global scholars have extensively utilized machine learning to aid in judgment and decision making by utilizing various sources of information, such as burn wound photos [25,26,27], patient demographics, vital signs, underlying diseases, total burn areas, and more [28,29]. Furthermore, the utilization of animal models for comparative prediction is extensively practiced across numerous prominent research institutions [30,31]. These advancements have significantly contributed to both basic research and clinical applications.

Moreover, we conducted a comprehensive comparison of our study with other similar research endeavors, which we have succinctly summarized in Table 7.

Based on the above comparison, it can be observed that our predictive model performs better in estimating the extended length of hospital stays. However, when it comes to predicting mortality/survival and complications, both our predictive method and Frye et al.’s method [28] have their own merits. This difference in estimation is likely due to slight variations in the extraction of clinical information and analysis methods.

In accordance with the achievements of previous studies, we have envisioned extracting a wider range of patient data and have employed seven machine-learning methods to generate more detailed predictions for various outcomes. By integrating this approach with the hospital information system (HIS) [32], our research not only assists clinical healthcare professionals in their decision-making processes but also provides objective references for explaining medical conditions to and predicting outcomes for patients and their families.

The clinical advantages of integrating our system with the hospital information system (HIS) lie in the enhanced convenience and efficiency of medical record and nursing record management. This integration enables the direct retrieval of computational results and medical staff–patient communication logs, effectively reducing the time spent on documentation tasks. Consequently, it leads to improved operational efficiency.

We acknowledge that our study is subject to certain limitations that warrant careful consideration. Firstly, the data for our analysis were exclusively sourced from a singular burn care unit located in Tainan, Taiwan. To enhance the robustness and generalizability of our findings, it is imperative that subsequent investigations incorporate data from a broader range of medical facilities. Secondly, it is plausible that certain granular data points may not have been collected in their entirety, underscoring the potential for additional insights. Thus, it becomes evident that further endeavors are essential to refine this model and elevate its overall performance.

## 5. Conclusions

This study aimed to develop a versatile machine-learning model to aid physicians in diagnosing disease progression and predicting the risk of death for burn patients. The model utilized a combination of patients’ basic health indicators, comorbidity indicators, and specific laboratory data as features. Additionally, we have successfully implemented and seamlessly integrated a web-based predictive application into the existing hospital information system (HIS) without requiring complex computational operations. This integration was well-received by physicians during the initial usage phase, indicating a high level of acceptance. We firmly believe that utilizing machine-learning algorithms to predict adverse outcomes in burn patients is a promising research approach that can assist physicians in promptly assessing disease severity following hospital admission. This early assessment enables them to select the most suitable and personalized treatment strategies, thereby improving patient prognosis.

Furthermore, in addressing the dynamic fluctuations in injury conditions, we can harness objective data acquisition to facilitate AI assistance in comprehensively interpreting information and subsequently delivering it to clinical caregivers and patients. This approach enables us to respond more promptly to changes in the patient’s medical condition.

For future studies, researchers can consider incorporating additional potential variables and conducting a feature selection process to enhance the quality of the models. With the continual emergence of novel physiological monitoring tools and laboratory diagnostic instruments, we anticipate the ability to gather a greater volume of valuable data for utilization. Simultaneously, the accumulation of clinical cases through real-world usage will further enhance the system’s performance in future operations.

## Figures and Tables

**Table 1:** Demographics—patients with graft surgery.

Feature	Overall	Graft Surgery		*p*-Value
		No	Yes	
	N = 224	N = 124	N = 100	
Age, mean (SD)	45.8 (20.3)	46.1 (20.8)	45.4 (19.9)	0.813
Gender_Female, n (%)	76 (33.9)	42 (33.9)	34 (34.0)	1
Gender_male, n (%)	148 (66.1)	82 (66.1)	66 (66.0)	
Burned area rank 1 (area <10%), n (%)	113 (50.4)	90 (72.6)	23 (23.0)	<0.001
Burned area rank 2 (area 10–19%), n (%)	24 (10.7)	4 (3.2)	20 (20.0)	
Burned area rank 3 (area 20–29%), n (%)	45 (20.1)	19 (15.3)	26 (26.0)	
Burned area rank 4 (area >30%), n (%)	42 (18.8)	11 (8.9)	31 (31.0)	
BMI (body mass index), mean (SD)	24.5 (4.8)	24.3 (4.4)	24.7 (5.3)	0.546
GPT (glutamate pyruvate transaminase), mean (SD)	38.7 (95.2)	43.0 (124.6)	33.2 (32.5)	0.4
WBC (white blood cell), mean (SD)	11.5 (6.3)	10.3 (5.0)	12.9 (7.5)	0.003
Hemoglobin, mean (SD)	14.4 (1.8)	14.2 (1.7)	14.6 (1.9)	0.081
Creatinine, mean (SD)	1.1 (1.1)	1.1 (1.2)	1.1 (0.9)	0.988
Smoking, n (%)	32 (14.3)	16 (12.9)	16 (16.0)	0.641
Burned perineum, n (%)	37 (16.5)	22 (17.7)	15 (15.0)	0.713
Burned limb, n (%)	62 (27.7)	42 (33.9)	20 (20.0)	0.031
Underwent escharotomy, n (%)	27 (12.1)	7 (5.6)	20 (20.0)	0.002
Baux score, mean (SD)	69.2 (27.7)	64.2 (28.5)	75.4 (25.4)	0.002

Note. Significance testing approaches (*p* value): Chi-square test for categorical variables; *t*-test for continuous variables.

**Table 2:** Demographics—patients with prolonged hospital stay.

Feature	Overall	Prolonged Hospital Stay		*p*-Value
		No	Yes	
	N = 224	N = 95	N = 129	
Age, mean (SD)	45.8 (20.3)	45.8 (20.5)	45.8 (20.3)	0.994
Gender_Female, n (%)	76 (33.9)	36 (37.9)	40 (31.0)	0.351
Gender_male, n (%)	148 (66.1)	59 (62.1)	89 (69.0)	
Burned area rank 1 (area <10%), n (%)	113 (50.4)	77 (81.1)	36 (27.9)	<0.001
Burned area rank 2 (area 10–19%), n (%)	24 (10.7)	3 (3.2)	21 (16.3)	
Burned area rank 3 (area 20–29%), n (%)	45 (20.1)	7 (7.4)	38 (29.5)	
Burned area rank 4 (area >30%), n (%)	42 (18.8)	8 (8.4)	34 (26.4)	
BMI (body mass index), mean (SD)	24.5 (4.8)	23.8 (4.0)	25.0 (5.3)	0.044
GPT (glutamate pyruvate transaminase), mean (SD)	38.7 (95.2)	42.1 (139.7)	36.1 (37.7)	0.687
WBC (white blood cell), mean (SD)	11.5 (6.3)	10.1 (5.1)	12.5 (6.9)	0.002
Hemoglobin, mean (SD)	14.4 (1.8)	14.2 (1.6)	14.5 (1.9)	0.219
Creatinine, mean (SD)	1.1 (1.1)	1.0 (0.2)	1.2 (1.4)	0.033
Smoking, n (%)	32 (14.3)	12 (12.6)	20 (15.5)	0.679
Burned perineum, n (%)	37 (16.5)	16 (16.8)	21 (16.3)	1
Burned limb, n (%)	62 (27.7)	33 (34.7)	29 (22.5)	0.061
Underwent escharotomy, n (%)	27 (12.1)	5 (5.3)	22 (17.1)	0.013
Baux score, mean (SD)	69.2 (27.7)	62.6 (30.1)	74.1 (24.9)	0.003

Note. Significance testing approaches (*p* value): Chi-square test for categorical variables; *t*-test for continuous variables.

**Table 3:** Demographics—patients with overall adverse effects.

Feature	Overall	Overall Adverse		*p*-Value
		No	Yes	
	N = 224	N = 86	N = 138	
Age, mean (SD)	45.8 (20.3)	44.4 (20.4)	46.6 (20.3)	0.439
Gender_Female, n (%)	76 (33.9)	33 (38.4)	43 (31.2)	0.335
Gender_male, n (%)	148 (66.1)	53 (61.6)	95 (68.8)	
Burned area rank 1 (area <10%), n (%)	113 (50.4)	76 (88.4)	37 (26.8)	<0.001
Burned area rank 2 (area 10–19%), n (%)	24 (10.7)	3 (3.5)	21 (15.2)	
Burned area rank 3 (area 20–29%), n (%)	45 (20.1)	6 (7.0)	39 (28.3)	
Burned area rank 4 (area >30%), n (%)	42 (18.8)	1 (1.2)	41 (29.7)	
BMI, mean (SD)	24.5 (4.8)	23.6 (4.0)	25.0 (5.2)	0.027
GPT, mean (SD)	38.7 (95.2)	28.4 (17.9)	45.1 (120.1)	0.111
WBC, mean (SD)	11.5 (6.3)	9.3 (4.0)	12.8 (7.1)	<0.001
Hemoglobin, mean (SD)	14.4 (1.8)	14.1 (1.5)	14.5 (1.9)	0.091
Creatinine, mean (SD)	1.1 (1.1)	0.9 (0.2)	1.2 (1.3)	0.014
Smoking, n (%)	32 (14.3)	11 (12.8)	21 (15.2)	0.758
Burned perineum, n (%)	37 (16.5)	14 (16.3)	23 (16.7)	1
Burned limb, n (%)	62 (27.7)	33 (38.4)	29 (21.0)	0.008
Underwent escharotomy, n (%)	27 (12.1)	1 (1.2)	26 (18.8)	<0.001
Baux score, mean (SD)	69.2 (27.7)	56.2 (22.8)	77.3 (27.5)	<0.001

Note. Significance testing approaches (*p* value): Chi-square test for categorical variables; *t*-test for continuous variables.

**Table 4:** Model performance.

**Model**	**Algorithm**	**Accuracy**	**Sensitivity**	**Specificity**	**AUC**	**AUC 95%CI**
Graft surgery	Random forest	0.765	0.833	0.711	0.756	0.639–0.874
	Logistic regression	0.706	0.7	0.711	0.755	0.638–0.873
	LightGBM	0.706	0.733	0.684	0.745	0.625–0.864
	XGBoost	0.721	0.733	0.711	0.738	0.616–0.859
**Model**	**Algorithm**	**Accuracy**	**Sensitivity**	**Specificity**	**AUC**	**AUC 95%CI**
Prolonged hospital stay	XGBoost	0.779	0.795	0.759	0.815	0.710–0.920
	Random forest	0.794	0.795	0.793	0.801	0.690–0.912
	LightGBM	0.706	0.769	0.621	0.797	0.688–0.905
	Logistic regression	0.676	0.718	0.621	0.720	0.595–0.844
**Model**	**Algorithm**	**Accuracy**	**Sensitivity**	**Specificity**	**AUC**	**AUC 95%CI**
Overall adverse effects	LightGBM	0.779	0.786	0.769	0.845	0.751–0.939
	Logistic regression	0.765	0.714	0.846	0.832	0.734–0.929
	XGBoost	0.765	0.786	0.731	0.822	0.724–0.921
	Random forest	0.765	0.738	0.808	0.816	0.716–0.916

**Table 5:** Comparisons of AI models and Baux score models.

Outcome	Model	Accuracy	Sensitivity	Specificity	AUC	AUC95%CI	DeLong Test (*p*)
Graft surgery	AI model (random forest)	0.765	0.833	0.711	0.756	0.639–0.874	0.206
	Baux score	0.574	0.433	0.684	0.641	0.509–0.774	
Prolonged hospital stay	AI model (XGBoost)	0.779	0.795	0.759	0.815	0.710–0.920	0.023
	Baux score	0.588	0.487	0.724	0.657	0.515–0.800	
Overall adverse effects	AI model (LightGBM)	0.779	0.786	0.769	0.845	0.751–0.939	0.008
	Baux score	0.559	0.524	0.615	0.619	0.484–0.754	

**Table 6:** A comparison with related studies.

Study	This Study	[22]	[23]	[24]
Sample size	224	66,611	6059	3264
Types of samples	High-risk patient admitted to our burn center	Population-based (England and Wales) case-cohort registry	Image in majority Patient data in minority	Image Animal models Simulated patient data
Outcome	Prolonged hospital stay (>14 days) Skin graft needed Adverse complications including mortality	Mortality	(%TBSA) Fluid estimations Burn depth Need for surgery Scarring	(%TBSA) Fluid requirements Burn depth Surgical candidacy
Study method	Seven machine-leaning methods	Four machine-leaning methods	Systemic review	Systemic review
Real-world implementation	Yes A predictive application with AI models was implemented and integrated into the existing HIS	N/A	N/A	N/A
Input data	Fourteen patient demographic features, TBSA, burned part, vital signs, laboratory results, comorbidities	Age, TBSA, type of burn, comorbidities	Vital signs Burn photos TBSA Inhalation TBSA	Simulated patient data 2D image Animal models
Testing results (AUC)	Prolonged hospital stay (>14 days) (0.795–0.811)	Mortality (0.945)	Burn depth (0.83)	Burn depth (0.662–0.925)
	Skin graft needed (0.788)			Surgery determination (0.793–1.000)
	Occurrence of adverse complications (0.872)			
Year	2023	2015	2015	2021

**Table 7:** A comparison with similar studies.

Study	This Study	[28]	[32]
Patient number	224	1585	1080
Types of patient origin	High-risk patient admitted to our burn center	Burn center inpatient in a medical center in the US	A regional burn center inpatient
Outcome	Prolonged hospital stay (>14 days) Skin graft needed Adverse complications including mortality	LOS (length of hospital stay) Survival	LOS (length of hospital stay)
Study method	Seven machine-leaning methods	An artificial neural network	Two machine-leaning methods
Real-world implementation	Yes A predictive application with AI models was implemented and integrated into the existing HIS	N/A	N/A
Input data	Fourteen patient demographic features, TBSA, burned part, vital signs, laboratory results, comorbidities	TBSA, burned part, type of transportation, burn mechanism	Sixteen patient demographic features, TBSA, burned part, vital signs, laboratory results, comorbidities, operation, burn depth
Testing results (AUC)	Prolonged hospital stay (>14 days) (0.795–0.811)	LOS (length of hospital stay) (0.72)	LOS (length of hospital stay) (0.487–0.718)
	Occurrence of adverse complications including mortality (0.872)	Survival (0.98)	
	Skin graft needed (0.788)		
Year	2023	1996	2010

## Data Availability

The dataset used for this study is available upon request from the corresponding author.
